# GCR1 and GPA1 coupling regulates nitrate, cell wall, immunity and light responses in *Arabidopsis*

**DOI:** 10.1038/s41598-019-42084-2

**Published:** 2019-04-09

**Authors:** Navjyoti Chakraborty, Kostya Kanyuka, Dinesh Kumar Jaiswal, Abhineet Kumar, Vivek Arora, Aakansha Malik, Neha Gupta, Richard Hooley, Nandula Raghuram

**Affiliations:** 1 University School of Biotechnology, G.G.S. Indraprastha University, Sector 16C, Dwarka, New Delhi 110078 India; 2 School of Basic and Applied Sciences, Maharaja Agrasen University, Baddi, Distt. Solan, Himachal Pradesh 174103 India; 3 Biointeractions and Crop Protection Department, Rothamsted Research, Harpenden, Hertfordshire, AL5 2JQ UK; 4 Department of Biology and Biochemistry, University of Bath, Claverton Down, Bath, BA2 7AY UK

## Abstract

G-protein signaling components have been attributed many biological roles in plants, but the extent of involvement of G-protein coupled receptor 1 (*GCR1*) with the Gα (*GPA1*) remained unknown. To address this, we have performed transcriptomic analyses on *Arabidopsis gpa1-5gcr1-5* double mutant and identified 656 differentially expressed genes (DEGs). MapMan and Gene Ontology analyses revealed global transcriptional changes associated with external stimulus, cell wall organization/biogenesis and secondary metabolite process among others. Comparative transcriptomic analyses using the single and double mutants of *gcr1-5* and *gpa1-5* identified 194, 139 and 391 exclusive DEGs respectively, whereas 64 DEGs were common to all three mutants. Further, pair wise comparison of DEGs of double mutant with single mutants of *gcr1-5* or *gpa1-5* showed about one-third and over half common DEGs, respectively. Further analysis of the DEGs exclusive to the double mutant using protein-protein interaction networks revealed molecular complexes associated with nitrate and light signaling and plant-pathogen interactions among others. Physiological and molecular validation of nitrate-response revealed the sensitivity of germination to low N in the double mutant and differential expression of nitrate transporter (and nitrate reductase in all three mutants). Taken together, *GCR1* and *GPA1* work in partnership as well as independently to regulate different pathways.

## Introduction

Heterotrimeric G-proteins regulate diverse signaling events in plants, following the dissociation of heterotrimer into GTP-bound Gα subunit and Gβγ dimers, which further activate the various downstream effectors for the coordinated regulation of plant responses. The model dicot *Arabidopsis* has been so far found to have only one α (GPA1), one β (AGB1), three γ subunits (AGG1-3), and three extra-large Gα proteins (XLG1-3)^1,2^. It has been shown that heterotrimeric G-proteins regulate cell growth and development, hormonal signaling, nitrate reductase gene expression and response to both abiotic and biotic stresses^3–7^. The upstream components of plant G-protein signalling and their interactions with G-proteins have been studied^8–10^ but still poorly understood. The best-considered GPCR candidate, *GCR1* in *Arabidopsis*, has been implicated in the regulation of DNA synthesis^11^, abolishing seed dormancy, reducing flowering time^12^, brassinosteroid and gibberellin-regulation of seed germination^13^, drought stress, ABA response, regulation of stomatal apperture^14^, blue light response^15^ and most recently in biotic stress, flavonoid biosynthesis, cytokinin biosynthesis, salicylic acid and ethylene response, and phosphate starvation^16^. Transcriptome analyses of *gpa1-5* has also identified DEGs involved in similar pathways including flavonoid biosynthesis, transcription factors, transporters and nutrient responses to nitrate and phosphate^17^.

The demonstration of self-activation of GPA1^18^, lack of a confirmed GPCR and its ligand or guanine nucleotide exchange factor (GEF) activity in plant GCR so far^19^ and the disagreement^20^ over the reported interaction between GCR1 and GPA1 in *Arabidopsis*^14,21^ were used to question the existence and the role of GPCRs in plant G-protein signalling^20^. Instead, it has been shown with the help of crystal structure and *in vitro* experiments that plant Gα-proteins are self-activating and spontaneously exchange GDP with GTP without the need of GEF activity^18,22^. The sustained activation of G-protein signaling occurs by endocytosis of the regulator of G-protein signalling (RGS1) in *Arabidopsis*^23^. The seven transmembrane RGS proteins were initially thought to be absent in most studied grasses and monocots^24^ but later it was found that RGS proteins are present in many grasses with frequent losses in different species like rice^25^. Moreover, the argument regarding the lack of heterotrimeric G-proteins in green algae (which are predicted to have GCRs) has been countered recently by the discovery of a complete G-protein complex in a green alga, *Chara braunii*^26^. Most recently, transcriptome analyses on *gcr1-5* mutant revealed differentially expressed genes belonging to known G-protein regulated processes^16^ suggesting the need to revisit the role of GCR1 in plant signalling in general and G-proteins in particular.

Till the GEF activity for GCR1 and its GPCR properties are proven, an overlap between the genes/processes/responses between the single mutants of *GCR1*^16^ and *GPA1*^17^ remains the best genetic evidence in favour of their functional association. This can be best validated by transcriptomic analyses of a double mutant, in comparison with either of the single mutants. The above two single mutant studies were done in the WS ecotype, whereas the double mutant isolated elsewhere was in the Col-0 ecotype^11^ and therefore, a double mutant in WS ecotype was necessary to validate the predictions made using the single mutants^16,17^. Accordingly, in this study, we used the *GPA1* and *GCR1* double mutant generated in WS background for whole transcriptome microarray analysis and comparison with single mutant data to demonstrate their combinatorial roles for various cellular responses and sensitivity of its seed germination to low nitrate.

## Results

### Characterization of the *gpa1-5gcr1-5* double mutant

**Figure 1:**
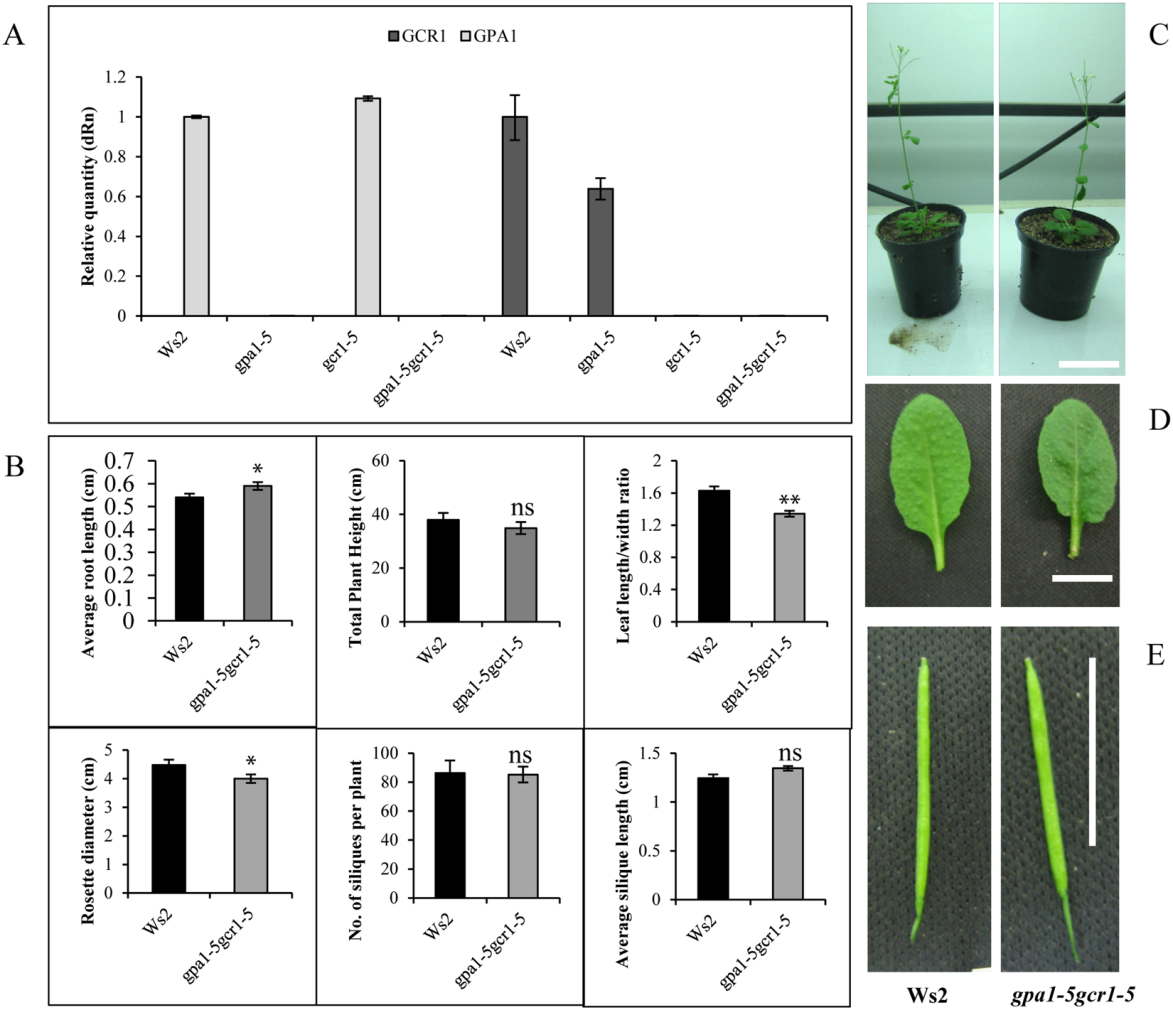
Characterization of the *gpa1-5gcr1-5* double mutant. (**A**) The mutants and WT were grown for 23 days and subjected to total RNA isolation and qRT-PCR to confirm the lack of expression of GPA1 or GCR1 in the single as well as double mutants. The data represent averages of three independent replicates ± SE. (**B**–**E**) Phenotypic characterization of the *gpa1-5gcr1-5* mutants. The double mutant and the WT were grown for 5 days on agar plates for root length comparison and were subsequently transferred to pots and grown to complete their life cycle to evaluate other phenotypic parameters shown. Each experiment was performed twice independently and the data represent averages of 10 individual plants ± SE (*P < 0.05, **P < 0.01 according to unpaired t-test using GraphPad Prism). The photographic strip of the Ws2 control have been reproduced from our previous paper^16^ under creative commons attribution license for ready reference. Scale bar = 1.0 cm.

A double mutant of *gpa1-5gcr1-5* was generated by crossing their confirmed single null mutants^16,17^ but its characterization was not reported earlier^6^. The null double mutant, devoid of expression of both *GPA1* and *GCR1*, was confirmed by qPCR (Fig. 1A). The mutant plants were phenotypically characterized for root length, plant height, leaf shape and other phenotypic traits. It was found that *gpa1-5gcr1-5* is similar to the only other known *gpa1gcr1* double mutant in Col-0 background^11^ with longer roots, less plant height, longer siliques, and rounded leaves and smaller rosette (Fig. 1B–E). Overall, the double mutant *gpa1-5gcr1-5* was found to be phenotypically closer to the *gpa1-5* single mutant^17^ than the *gcr1-5* single mutant^16^, though in most cases, the phenotype is somewhere between the two single mutants.

### Microarray analysis and validation

**Figure 2:**
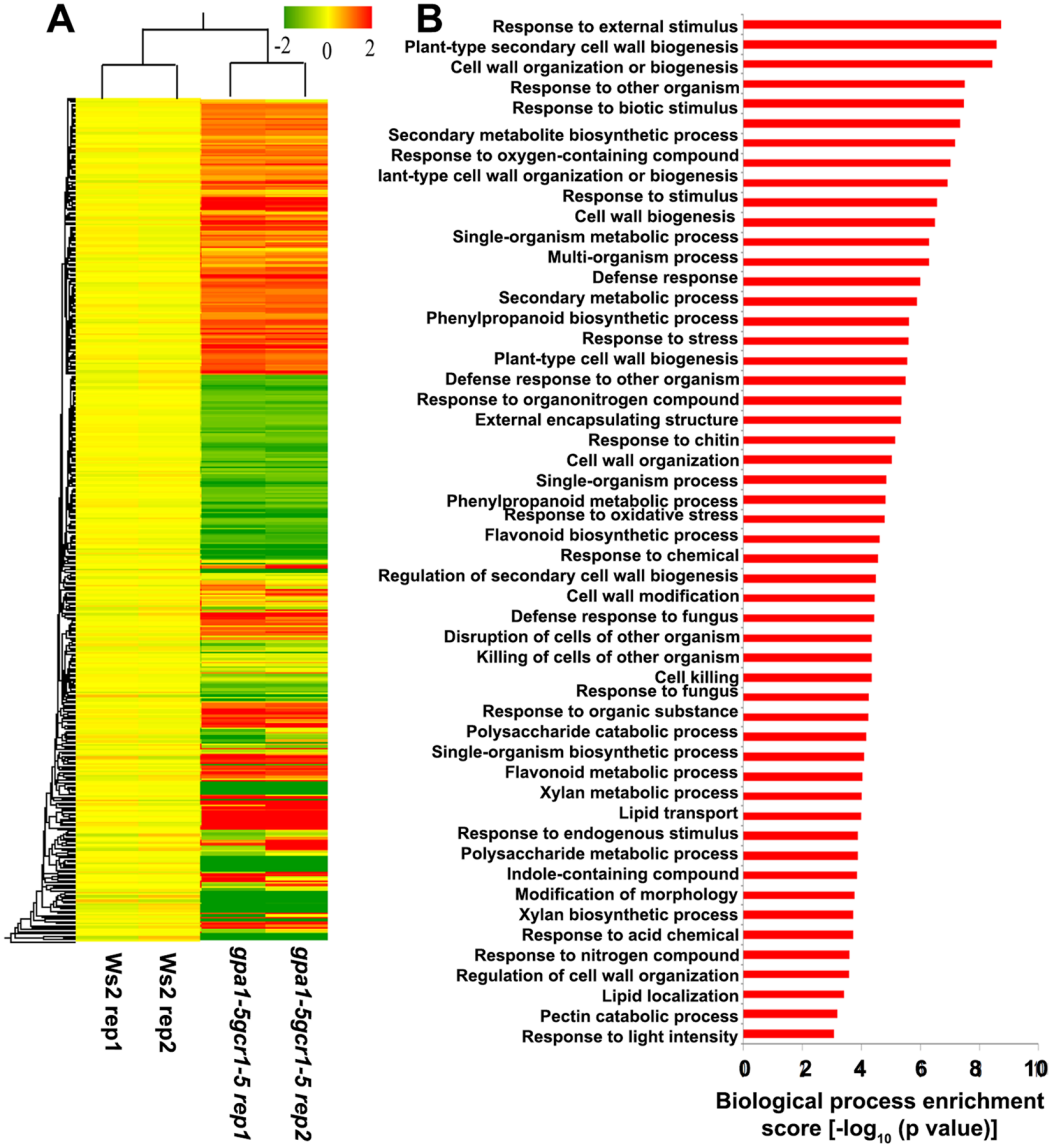
(**A**) Heat map and GO analyses of differentially expressed genes. The background-subtracted, p-value filtered and geo-mean cutoff microarray data of the double mutant was subjected to hierarchical clustering using Genespring software ver. 11.5 to generate the heat map. Red, green and yellow represent up-regulated, down–regulated and unregulated genes, respectively. (**B**) The DEGs were functionally categorized into various biological processes using AgriGO2.0 tool. The p-values of biological processes were log transformed (−log_10_) and plotted (**B**). The complete results of AgriGO analyses, which include p-value, FDR and the numbers of DEGs associated with each biological process are listed in the supplementary Table S3.

**Figure 3:**
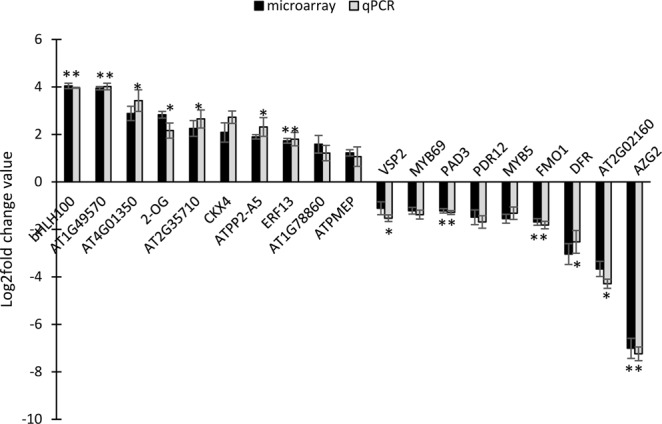
qPCR validation of differentially expressed genes in *gpa1-5gcr1-5* double mutant. A total of 19 DEGs (10 up- and 9 down-regulated) were selected and subjected to RT-qPCR. The experiment was carried out using three biological replicates and the values are presented as log_2_FC ± SE. qPCR was performed in triplicate and the ratios of statistics were calculated relative to the internal control gene *Actin2* (*P < 0.05, **P < 0.01 vs. control).

The MIAME compliant microarray replicates had high correlation coefficient (>0.9), clearly indicating the robustness and a high level of reproducibility of the data (Table S1). The Benjamini Hochberg FDR procedure at a cut-off value of p ≤ 0.05 was used for multiple testing corrections. A stringent cut-off value of 1.0 (geometric mean log_2_) with a p-value of ≤0.05 was used to identify 829 differentially regulated transcripts in the double mutant (422 up-regulated and 407 down-regulated). These transcripts corresponded to 656 unique differentially expressed genes (DEGs), 306 up-regulated and 350 down-regulated. A list of 10 most up- and down-regulated genes is shown in Table 1 and the heat map of all the DEGs and their GO classification is shown in Fig. 2. In order to validate the microarray results, 19 DEGs (10 up- and 9 down-regulated) were selected spanning each of the important functional categories and subjected to RT-qPCR using gene specific primers tested for efficiency (100 ± 10%). The list of these genes and the primer sequences used are given in the Table S2. The results of RT-qPCR matched with the microarray data in all the cases (Fig. 3) with Pearson’s product moment correlation of >0.99 (p-value = 6.54E-17), validating the basic trends of regulation of gene expression found in the microarray analyses.

**Table 1:** List of top 10 each up-regulated and down-regulated DEGs in the *gpa1-5gcr1-5* mutant.

Locus id	Accession id	Gene name	Log2FC	p-value
**Up-regulated in** ***gpa1-5gcr1-5***				
AT3G04330	NM_111304	Kunitz family trypsin and protease inhibitor protein	6.20	0.0127
AT1G63580	NM_105036	Receptor-like protein kinase-related family protein	5.31	0.0341
AT1G65570	NM_105231	Pectin lyase-like superfamily protein	5.30	0.0493
AT5G11140	NM_121152	*Arabidopsis* phospholipase-like protein (PEARLI 4) family	4.99	0.0003
AT3G01580	NM_111024	Tetratricopeptide repeat (TPR)-like superfamily protein	4.94	0.0463
AT3G55550	NM_115412	LECRK-S.4	4.91	0.0002
AT4G15650	NM_117656	unknown protein	4.54	0.0466
AT2G06002	NR_022465	ncRNA	4.45	0.0025
AT5G35300	NM_122921	unknown protein	4.12	0.0082
AT2G41240	NM_129689	BHLH100	4.04	0.0067
**Down-regulated in** ***gpa1-5gcr1-5***				
AT1G04890	NM_100367	Protein of unknown function DUF593	−8.77	0.000
AT2G38900	NM_129447	PR (pathogenesis-related) peptide	−7.53	0.001
AT3G25170	NM_113422	RALFL26	−7.35	0.024
AT5G47350	NM_124106	Alpha/beta-Hydrolases superfamily protein	−7.11	0.003
AT5G50300	NM_124409	AZG2	−7.01	0.011
AT4G15750	NM_117666	Plant invertase/pectin methylesterase inhibitor superfamily protein	−6.58	0.000
AT5G10880	NM_121126	tRNA synthetase-related/tRNA ligase-related	−6.35	0.006
AT4G40100	NM_120176	PRSL1	−5.87	0.028
AT3G58190	NM_115681	LBD29	−5.33	0.011
AT3G24510	NM_113361	Defensin-like (DEFL) family protein.	−5.33	0.004

### Gene Ontology and MapMan pathway analyses of double mutant DEGs

**Figure 4:**
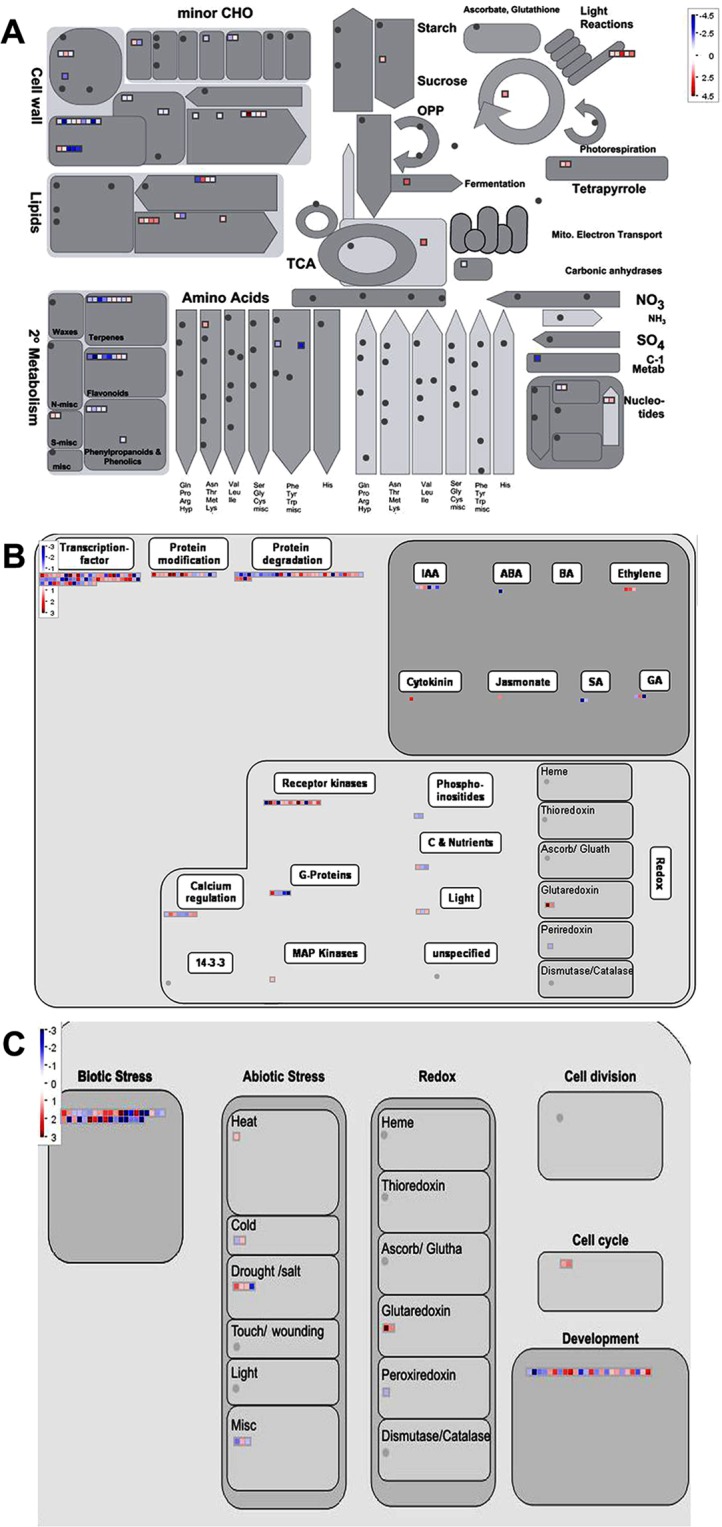
Mapping of DEGs found in the *gpa1-5gcr1-5* double mutant into various pathways using MapMan. (**A**) DEGs mapped into metabolic pathways. (**B**) DEGs associated with regulation. (**C**) DEGs assigned to cellular responses. Each box represents a DEG while the red and blue colours indicate up- and down-regulated DEGs, respectively.

To understand the biological effects of loss of both GCR1 and GPA1 function, we performed the GO analyses of the DEGs identified in the double mutant using AgriGO2.0 tool^27^. The statistically overrepresented GO terms (based on p value and FDR) were considered for further analyses (Fig. 2B and Table S3). All the 656 DEGs were broadly classified into biological processes, molecular functions and cellular components. The over-represented GO terms for biological processes were “response to external stimulus”, “plant-type secondary cell wall biogenesis”, “cell wall organization or biogenesis”, “response to external biotic stimulus”, “response to other organism”, “response to biotic stimulus” and secondary metabolite biosynthetic process” among others. In molecular function category, we observed the significant GO terms were “terpene synthase activity”, “O-methyltransferase activity”, “carbon-oxygen lyase activity”, “acting on phosphates tetrapyrrole binding” and “transcription factor activity” among others whereas “extracellular region” GO term identified for cellular component (Table S3). We also mapped these DEGs into various pathways using MapMan^28^. Comparative analyses showed a high degree of agreement between GO terms and MapMan pathways. The DEGs were broadly mapped into various pathways (bins) such as metabolic processes (Fig. 4A), different levels of regulation (Fig. 4B) and cellular responses (Fig. 4C). Further insight into these pathways (sub-bins) showed that many DEGs were mapped into biotic and abiotic stress pathways, development, cell wall, lipid and amino acid metabolism, hormone signaling, protein modification and degradation among others. Some DEGs were also classified as receptor like kinases (RLKs), transcriptional regulators and genes regulated by calcium and G-protein signaling (Fig. 4).

### Sub cellular distribution of DEGs and identification of associated transcription factors

**Figure 5:**
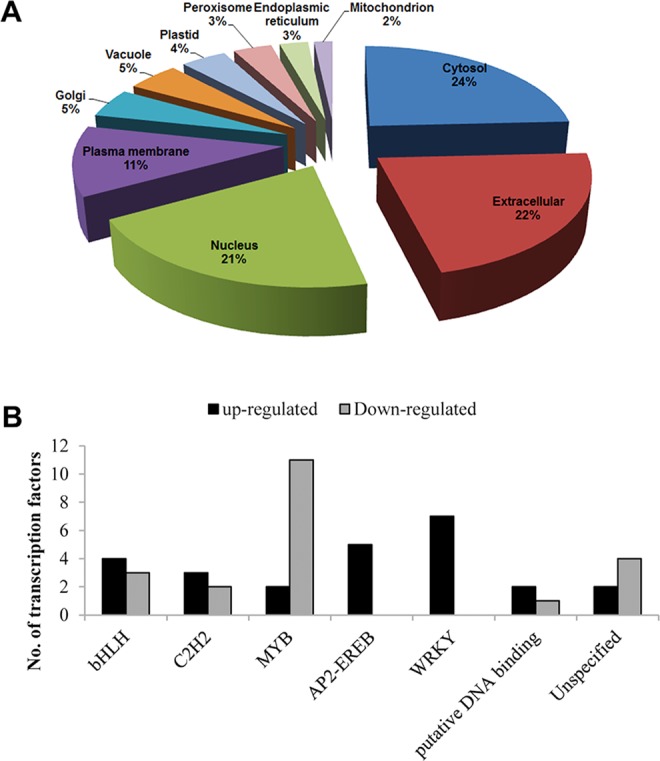
Subcellular localization of DEGs and classification of transcription factors among them. (**A**) Subcellular distributions of the DEGs identified in the double mutant as predicted using YLoc program. (**B**) Identification and classification of transcription factors among the DEGs in the double mutant using plantTFDB.

To understand the global cellular context of both GCR1 and GPA1 mutations in terms of the affected subcellular organelles and associated pathways, all the DEGs were subjected to subcellular prediction using YLoc program. We observed that majority fraction of the DEGs were distributed into cytosol (24%), extracellular (22%), nucleus (21%) and plasma membrane (11%) among others (Fig. 5A). This suggests that both GCR1 and GPA1 regulate many processes and pathways that operated within these organelles. Nuclear genome is an important target for myriad signaling pathways that culminate in gene regulation by transcription factors (TFs). A search using the DEGs at the plant transcription factor database (plantTFDB 2.0)^29^ revealed 64 transcription factors (Table S4) belonging to 22 families. Their regulation was nearly equally distributed in the double mutant, with 34 up-regulated and 30 down-regulated TF genes. (Table S4). Most of them belong to the class of bHLH, C2H2, MYB, WRKY and AP2-EREB families, other than putative and unspecified ones (Fig. 5B). While many of the MYB family members were found to be down-regulated in the double mutant, none of the transcription factors of AP2-EREB and WRKY families were down-regulated. On the other hand, in the bHLH and C2H2 families, the up- and down-regulated transcription factors showed a mixed distribution. Sixteen TFs were commonly regulated in *gpa1-5* while none of these TFs were common in the *gcr1-5* mutant. The guard cell functions and root differentiation are mediated through G-protein signaling^14^. The transcriptional regulators such as bHLH, MYB and WD40 are known to regulate these functions^30^ and many of these regulators were identified as DEGs in our datasets. MYB and WRKY belong to a major TFs class and were reported to be involved in stress responses^31,32^. Members of AP2/EREB class of TFs have been reported to be involved in storage compound, fatty acid biosynthesis and stress responses^33^. The up regulation of two TFs, bHLH100 and ERF13 and the down regulation of MYB69 and MYB5 were validated in the double mutant using qPCR (Figs S1 and S2).

### Double and single mutants share substantial number of DEGs

**Figure 6:**
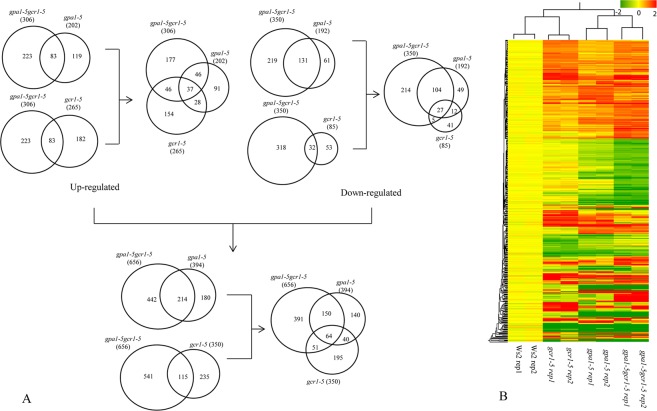
(**A**) Venn selection of differentially regulated genes between single and double mutants. The DEGs identified in the double mutant in the current study were compared with those identified earlier in the single mutants of *gpa1-5*^17^ and *gcr1-5*^16^ and shown as up/down regulated subsets or together. (**B**) Hierarchical clustering of DEGs obtained from all the 3 mutants shows that *gpa1-5gcr1-5* double mutant is closer to the *gpa1-5* mutant than the *gcr1-5* mutant.

In order to gain a comprehensive view of the differential regulation of the affected genes in the single and double mutants of *GPA1* and *GCR1*, we compared the DEGs obtained in *gpa1-5gcr1-5* double mutant to those of the single *gpa1-5*^17^ and *gcr1-5*^16^ mutants. Out of the 350 *GCR1*-regulated genes in the single mutant, 115 (or 34%) were common to the 656 DEGs in the double mutant. Similarly, out of the 394 of the *GPA1* regulated genes in the single mutant 214 (or 54%) were common to the 656 DEGs in the double mutant. Only 64 DEGs were found to be shared amongst all three mutants (Fig. 6A). The hierarchical clustering of the DEGs from all the mutants revealed that the double mutant (*gpa1-5gcr1-5*) is closer to *gpa1-5* and that *gcr1-5* is closer to the wild type (Fig. 6B). This closely parallels the similarity patterns in their phenotypes.

If the genetic interactions are additive, the genes differentially expressed in the double mutant should have been the sum of all the DEGs found in the single mutant. Also, all the DEGs shared by the single mutants should also have been common to the double mutant, but this was not observed using the log2 fold change (log2FC) cut-off of 1.0. The double mutant has almost double the number of DEGs identified in each of the single mutants. 64 DEGs (37 up-regulated; 27 down-regulated) were common to all the three mutants, while the single mutants shared 104 DEGs between them. A closer look at these 104 DEGs revealed that they did not light up in the double mutant due to either the stringent p-value cut-off of 0.05 or log2FC cut-off of 1.0. Out of the 28 such up-regulated genes that did not light up in the double mutant, 10 did not meet the p-value cut-off, 10 had log2FC value of 0.8 and above, while the remaining 8 genes had log2FC value of less than 0.8. Similarly, among the 12 such down-regulated genes, only 4 genes did not meet the log2FC cut-off of −1.0 and the rest did not make it to the list due to p-value cut-off of 0.05 despite having log2FC values beyond −1.0. We validated 10 DEGs from the list shared only by the single mutants and 10 DEGs unique to the double mutant by qPCR (Figs S1 and S2).

### Abiotic and biotic stress

Heterotrimeric G-protein-dependent immune regulation^34–37^ and abiotic stress-responses^4,38–40^ are well known in plants. Functional analyses of the DEGs revealed that “response to stimulus” constitutes the top most GO category of genes regulated by *GPA1* and/or *GCR1*. Among these DEGs, 32 genes (including *ESC*, *ARR22*, and *TT7*) were reported to be *GPA1*-regulated^17^, while 23 (including *CAD1*, *EF1α*, and *WRKY 53*) of them have been reported to be regulated by *GCR1*^16^, which also includes the 15 genes that are regulated by both *GPA1* and *GCR1*. The genes that are regulated by both *GPA1* and *GCR1* include *Arabidopsis thaliana* phloem protein 2 A5 (*ATPP2-A5*), dark inducible 11 (*DIN11*), phytoalexin deficient 3 (*PAD3*), etc. Many DEGs such as *DIN11*, *FMO1*, *MEE16*, and *PAD3* have been reported to be differentially regulated in *gpa1-5*^17^ and *gcr1-5* mutants^16,41^. These include several well-known stress-responsive genes such as low temperature induced 78 (*LTI78*), plant defensin 2.5 (*PDF2*.*5*), ethylene response factor (*ERF6*), and several peroxidases and transcription factors. Analysis of the DEGs using MapMan revealed them to be involved in abiotic stresses such as cold, heat, drought, salt etc., as well as in biotic stresses. More detailed mapping revealed that 225 out of total 656 DEGs belong to the biotic stress category (Fig. S3), though a few of them are also involved in abiotic stress. A majority of these 225 genes were mapped into signalling, proteolysis, cell wall, PR-proteins and secondary metabolites. The basic trends of their regulation in the mutant have been confirmed by qRT-PCR on two up-regulated genes (peroxidise family protein gene (AT1G49570) and ATPP2A5) and two down-regulated ones (*PDR12* and *PAD3*), as shown in Fig. 3.

### Secondary metabolism

The GO class associated with secondary metabolites were found to be an important category, so we checked the involvement of *GCR1*/*GPA1* in regulating the genes of secondary metabolism. We found that 107 DEGs belong to the biosynthesis of flavonoids and isoprenoids based on MapMan as well as pathway analysis using AraCyc database (Fig. S4, Table 2). The genes involved in flavonoid biosynthesis include 2-oxoglutarate, dihydroflavanol-4-reductase (*DFR*), UDP-glucosyl transferase 73C6 (*UGT73C6*), etc., while those involved in isoprenoid biosynthesis include dehydrodolichyl diphosphate synthase, myrcene synthase, terpene synthase 21 (*TPS21*), etc. The basic trends of their regulation in the mutants have been confirmed by qRT-PCR on the up-regulated gene 2-oxoglutarate and two down-regulated ones (*FMO1* and *DFR*), as shown in Fig. 3. Flavonoid biosynthesis was also found to be regulated in our previous studies using single mutants of *GPA1* and *GCR1*, but many more genes belonging to this category are differentially regulated in the double mutant. Thus, out of the 11 DEGs that regulate flavonoid biosynthesis found in the double mutant, only two were differentially regulated in both the single mutants, whereas 5 genes were differentially regulated in *gpa1-5* and 3 genes in *gcr1-5*.

**Table 2:** Secondary metabolite pathways identified in *gpa1-5*, *gcr1-5*, *gpa1-5gcr1-5* mutants. The significantly enriched pathways are represented in terms of p-value and shown in bold. The significantly enriched common pathways identified in all three mutants are marked with asterisk (*).

S. No.	Pathway name	p-value		
		*gpa1-5*	*gpa1-5gcr1-5*	*gcr1-5*
1	Monoterpene biosynthesis	0.122093	**9.5E-06**	NA
2	Gibberellin inactivation II (methylation)	**0.042435**	**0.000841**	NA
3	2,3-cis-flavanols biosynthesis	0.021443	**0.02924**	NA
4	Homogalacturonan degradation	0.099275	**0.002344**	**0.038723**
5	Leucodelphinidin biosynthesis*	**0.003796**	**0.011516**	**0.0322**
6	Leucopelargonidin and leucocyanidin biosynthesis*	**0.003796**	**0.011516**	**0.0322**
7	Camalexin biosynthesis	0.062988	0.08522	**0.027535**
8	Flavonol biosynthesis	**0.005997**	0.095354	0.154768
9	Coniferin metabolism	**0.006383**	0.163288	0.05435
10	Monolignol glucosides biosynthesis	**0.006383**	0.163288	0.05435
11	Flavonoid biosynthesis	0.164026	0.261912	**0.038214**
12	Superpathway of flavones and derivatives biosynthesis	**0.043732**	0.279198	0.44851

### Development

We also detected the association of 80 DEGs in developmental processes (Fig. 4). These genes include senescence-associated gene 12 (*SAG12*), vegetative storage protein 2 (*VSP2*), lateral organ boundaries-domain 29 (*LBD29*), several expansins, etc. Out of these, few genes like expansins are involved in cell wall modification. Genes like transparent testa 8 (*TT8*), tetratricopeptide repeat 3 (*TPR3*), cytokinin response factor 4 (*CRF4*), etc. are involved in development of shoot while transparent testa 16 (*TT*16), shatterproof 2 (*SHP2*), flowering locus T (*FT*), etc. are involved in flower development. *GPA1* has been previously reported^17^ to be involved in developmental processes and hence, shows a larger convergence with 17 genes being common between them. Only four DEGs were found to be common to *gcr1-5*^16^ in this category. We confirmed the basic trends of regulation in the mutant in this category using qPCR on the up-regulated (AT2G35710 and AT1G78860) as well as down-regulated (*VSP2* and AT2G02160) genes (Fig. 3).

### Hormone response

G-protein signaling has been implicated in regulation of hormone signaling in plants^41–43^. GO and MapMan analyses showed that 37 of the DEGs were associated with hormone biosynthesis and signaling (Figs 2 and 4). These include genes which are responsive to cytokinin, ethylene, ABA, auxin, salicylic acid, etc. Ethylene is known to down-regulate the expression of AGB1^44^ and the role of *GPA1* in ethylene signalling operated in guard cell is known^45^. Cytokinin oxidase 4 (*CKX4*) and cytokinin response factor 4 (*CRF4*) are involved in cytokinin biosynthesis/response; ethylene response factor 6 and 13 (*ERF6* and *ERF13*) and pleiotropic drug resistance 12 (*PDR12*) are involved in ethylene response. A few others like *MYB43*, hydroxysteroid dehydrogenase (*HSD1*), responsive to desiccation 26 (*RD26*), syntaxin of plants 121 (*SYP121*) etc., are involved in ABA response. A few auxin-responsive genes like *PDR12* and *LBD29* were also found among the hormone-responsive genes. Though hormone response was found as a major category in *gcr1-5*^16^, the overlap to the double mutant in terms of DEGs was limited to only 2 genes. Similarly, only 3 DEGs were found to be common to the *gpa1*^17^ and double mutant. The basic trends of their regulation in the double mutant have been confirmed by qRT-PCR on the up-regulated genes, *CKX4* and *ERF13*, as well as the down-regulated gene *PDR12*, as shown in Fig. 3.

### Transport

Twenty three genes related to transport were also found to be differentially regulated in the double mutant (Table S3). These include lipid transporters (*LPTs*), oligopeptide transporters (*POT*, *OPT5*), nuclear transport factor (*NTF2*), as well as nutrient transporters such as methylammonium transporter (*TIP 2;3*), phosphate transporter (*APT1*), nitrate excretion transporter (*NAXT1*) and high affinity K^+^ transporter (*HKT1*). A few of these DEGs have been reported earlier in other G-protein mutants^41,46^. The basic trends of their regulation in the mutant have been confirmed by qRT-PCR on the down-regulated genes *PDR12* and *AZG2*, as shown in Fig. 3. Interestingly, transport was also found to be a major response category in the transcriptomic analyses of the *gpa1-5* mutant^17^, but not in the *gcr1-5* mutant^16^.

### Cellular processes and molecular complexes regulated by both GCR1 and GPA1 function

**Figure 7:**
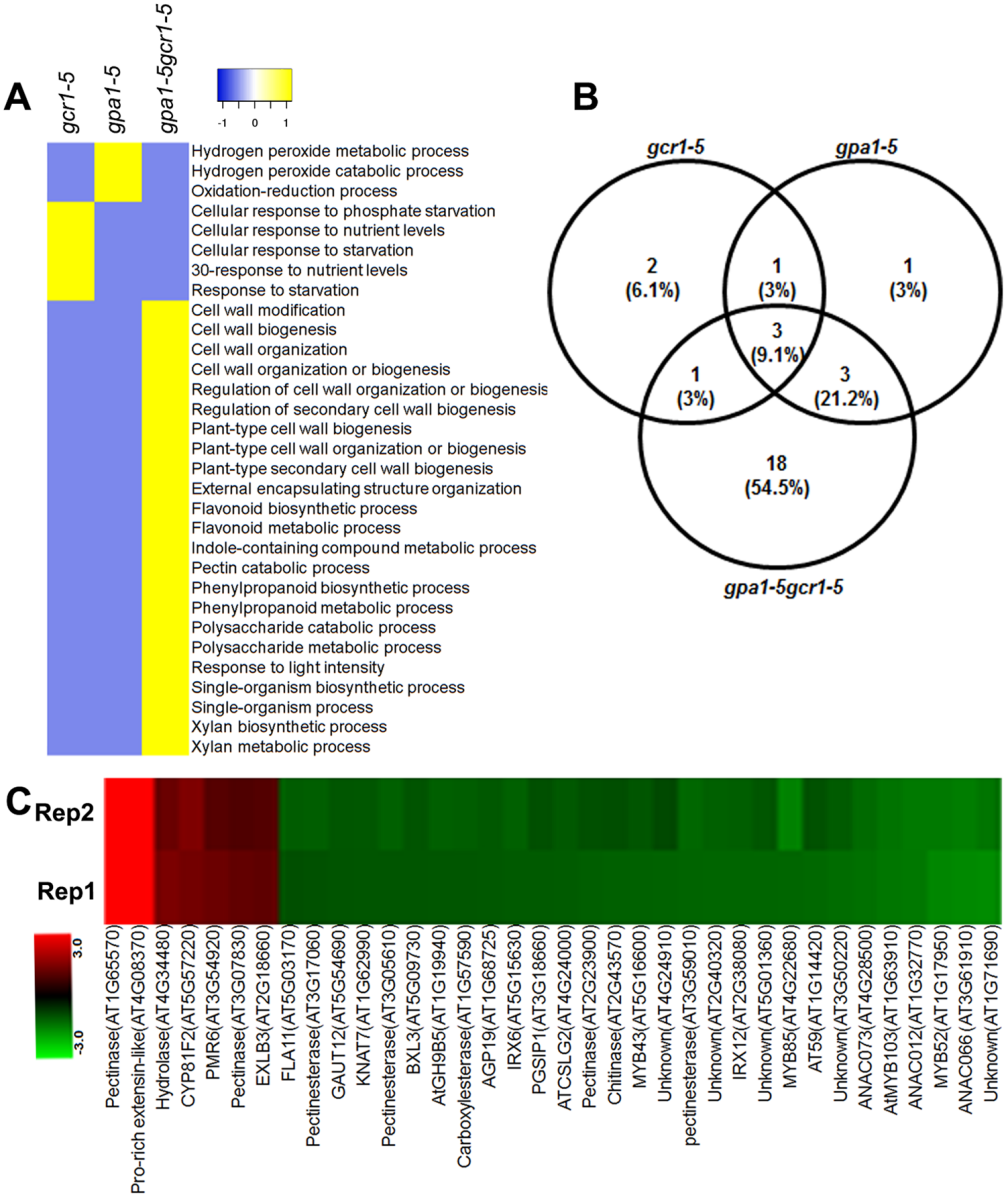
Heat map of biological processes exclusive to each of the three mutants and cell wall associated DEGs in the double mutant. The GO classes of DEGs exclusive to each of the single and double mutants were used for the analysis. (**A**) Heat map of the exclusive biological processes generated using heatmapper (http://heatmapper.ca/). The default colour scheme depicts the presence or absence of the exclusive GO classes as yellow or blue respectively. (**B**) Venn selection of cell wall associated DEGs from all three mutants identified by MapMan. (**C**) Heat map showing the cell wall associated exclusive DEGs identified in the double mutant using GO and MapMan analyses. Heat map was generated using Multi Experiment Viewer software (http://mev.tm4.org/#/welcome).

To understand the function of DEGs detected in the *gpa1-5gcr1-5* double mutant, we compared significantly overrepresented GO terms and observed both overlapping as well exclusive biological processes in all three mutant datasets (Table S5). The comparison clearly revealed that processes exclusive to the double mutant predominantly regulate cell wall composition and associated metabolic processes (Fig. 7A). MapMan analyses also revealed the over-representation of cell wall-associated DEGs in the double mutant (Fig. 7B). The results of both AgriGO (Fig. 7A) and MapMan analyses (Fig. 7B) are similar in the sense that the double mutant showed higher number of cell wall-associated exclusive DEGs as compared to either of the single mutants. A combination of both GO and MapMan analyses led to the identification of 36 cell wall-associated exclusive DEGs in the double mutant (Fig. 7C). Majority of these DEGs such as the family members of *ANAC*, *MYC*, *MYB* and pectinesterase were down-regulated, whereas pectinase, expansin-like B3 precursor, proline-rich extensin-like among others up-regulated in the double mutant. To validate the expression level of cell wall associated DEGs identified in the *gpa1-5gcr1-5* double mutant, 4 DEGs were selected for qPCR validation. Three DEGs, beta-xylosidase 3 (*BXL3*), COBRA-like 4 (*COBL4*), and galacturonosyl transferase 12 (*GAUT12*), were down-regulated, whereas pectin methylesterase (*ATPMEPCRB*) was up-regulated in the *gpa1-5gcr1-5* double mutant (Fig. S5), confirming their trend on the microarray. The BXL3 is generally localized in the extracellular matrix and is involved in the hydrolysis of arabinan, whereas COBL4, also known as irregular xylem 6 (IRX6), is involved in the secondary cell wall biosynthesis. The loss of function of *GAUT12*, also known as irregular xylem 8 (*IRX8*), significantly reduces xylose contents in the cell walls whereas ATPMEPCRB act on cell wall pectin in plant. The modulation of the expression of these genes in the double mutant indicates *GCR1* and *GPA1* coupling in the regulation of the cell wall.

**Figure 8:**
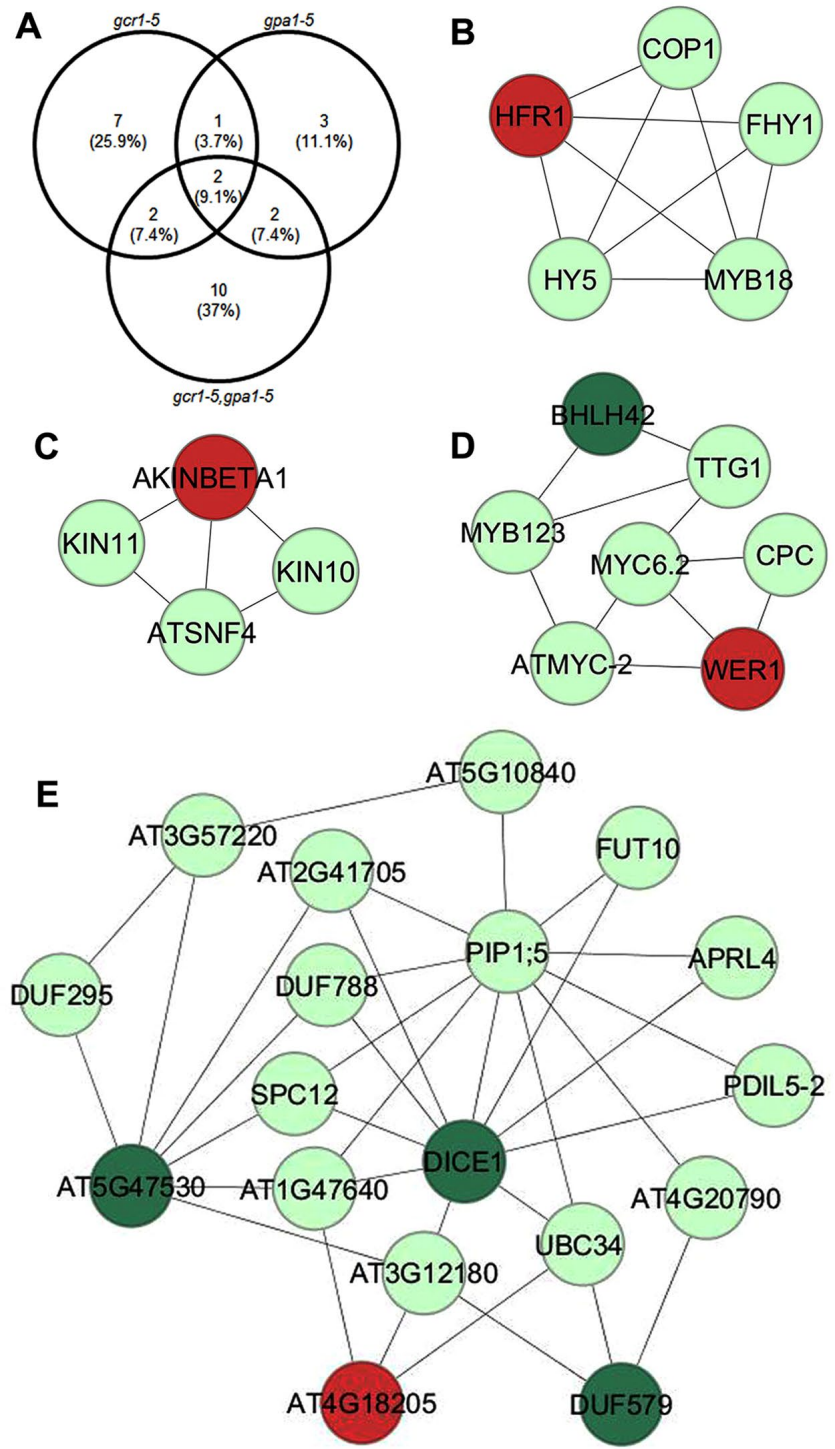
PPI networks of exclusive DEGs identified in the double mutant. (**A**) Venn diagram showing the overlapping and exclusive DEGs identified as interactors of G-protein signaling components. (**B**–**E**) The protein-protein interaction (PPI) networks were constructed with Cytoscape using experimentally validated interactions obtained from BioGRID and STRING databases. Sub-clustering of the PPI networks was performed using the MCODE plugin in Cytoscape and representative networks are shown. The red and dark green nodes represent the up-regulated and down-regulated DEGs, respectively. Interactors that are not among DEGs identified in the double mutant are assigned with light green colour.

To further understand the combinatorial role of GCR1 and GPA1 in cellular response, we used the DEGs from all three mutants to search in the G-protein interactome^47^, MIND database^48^, XLGs interactome^49^ and RGS1 protein networks^50^. We observed association with known G-protein signaling components in 12, 8 and 16 DEGs in the *gcr1-*5, *gpa1-5* and *gpa1-5gcr1-5* mutants, respectively (Fig. 8A). Only two DEGs namely phloem protein 2 A5 and methionine sulfoxide reductase B7 were found to be the common interactor DEGs among all three mutants (Table S6). To further delineate these complex regulations, we developed PPI networks of exclusive DEGs identified in the double mutant and mapped these DEGs into networks. To construct the PPI networks, we retrieved the experimentally validated interactions list from STRING and BioGRID databases and assigned the colour code to the nodes using DEGs expression value. The networks consisting of 2216 nodes and 3499 edges were analysed and viewed in Cytoscape 3.0.0^51^. The PPI network analyses showed many of the DEGs interacting with other components in the networks (Fig. 8B–E). Sub-clustering of the networks using MCODE plugin in Cytoscape revealed 7 highly connected molecular complexes/sub-clusters (Figs 8 and S6). Four molecular complexes having MCODE score >3 with node number >3 (Fig. 8B–E) were selected for further analyses. A total of 5, 18, 4, 7 nodes and 9, 35, 5, 11 edges were detected in sub-cluster 1, 2, 3 and 4, respectively. All seven sub-clusters details are mentioned in Table S7. The sub-cluster 1 includes transcriptional regulators associated with light signaling such as HY5 (Long Hypocotyl 5), COP1 (Constitutive Photomorphogenic1) and HFR1 (Long Hypocotyl in Farred1) (Fig. 8, Table S7). The sub-clusters 4 also include transcription regulators such as ATMYC-2, MYC6.2, ATMYB123, homeodomain-like superfamily protein involve in diverse biological processes. The miscellaneous interactors such as auxin-responsive family protein, glycosyl transferase family 4 protein, nucleotide-sugar transporter family protein, and ubiquitin-conjugating enzyme 34 among others as were identified in sub-cluster 2. AKINBETA1, KIN10, KIN11, and SNF4 genes were identified in sub-cluster 3 and these protein kinases are involved in various cell signaling process. The identification of DEGs in these molecular complexes suggests that associated cellular pathways may be regulated by the combined function of GCR1 and GPA1 in *Arabidopsis*.

### Germination of *gpa1-5gcr1-5* double mutant is sensitive to low nitrate

**Figure 9:**
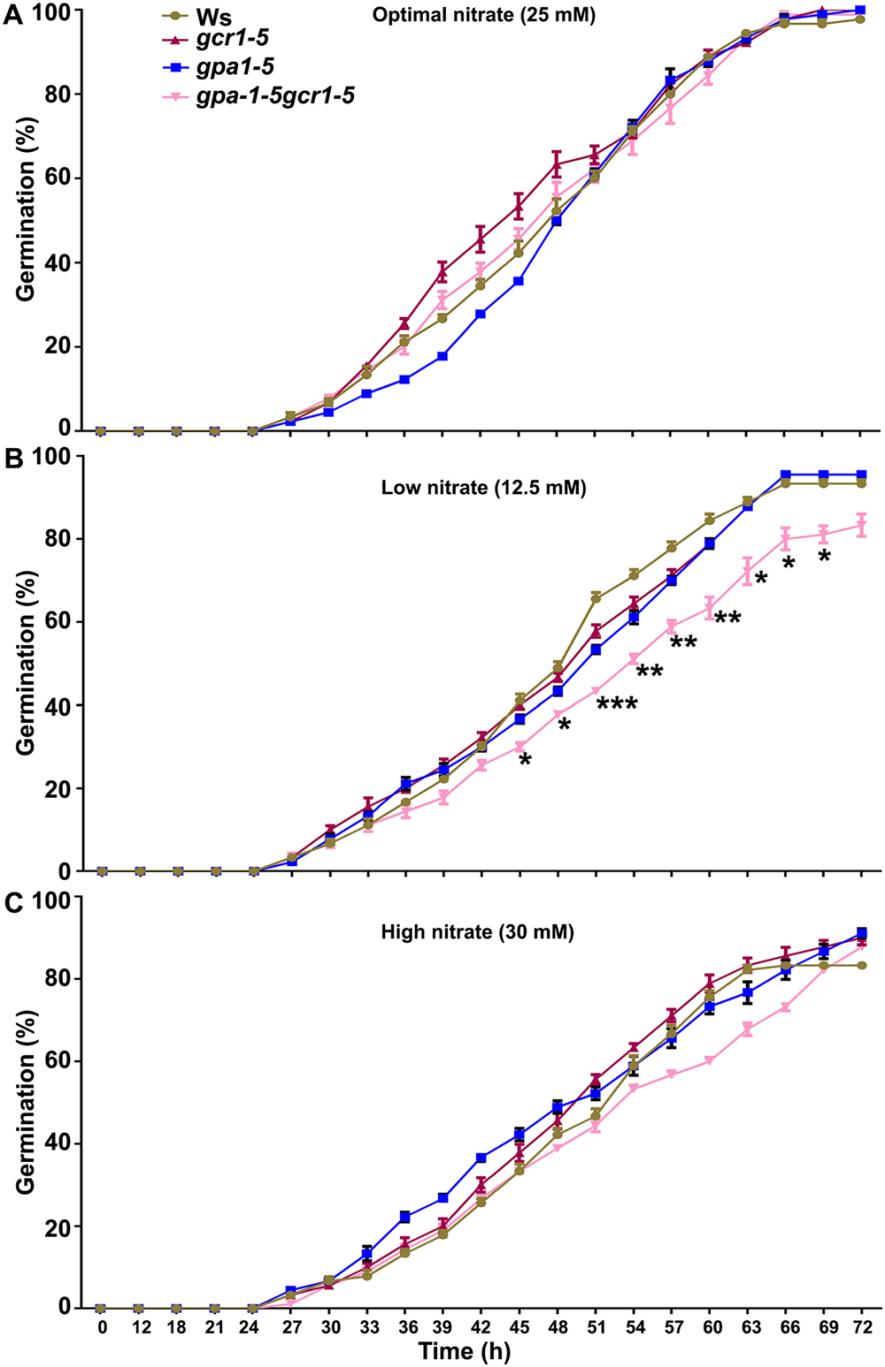
N-responsive germination in single and double mutants. Thirty seeds each of the wild-type (Ws2) and all three mutants, *gpa1-5*, *gcr1-5* and *gpa1-5gcr1-5,* were surface sterilized and stratified at 4 °C in dark for 48 h. These seeds were placed on 1X B5 agar plates supplemented with different concentrations of KNO_3_ as shown for optimal (**A**), low (**B**) and high (**C**) dose of nitrate. The plates were transferred to growth chambers maintained at 22 ± 1 °C and after 12 h germination was monitored at every 3 h until 72 h. The data are plotted as a percentage of germinated seeds along with standard error bars. The data was statistically analysed using ANOVA in the GraphPad Prism 6.0 (*P < 0.05, **P < 0.01, ***P < 0.001).

The effect of N and N-associated genes on seed germination is well known in plants^52–54^. We analysed the role of G-protein signaling on nitrate-responsive germination in single mutants (*gpa1-5*, *gcr1-5*) and their double mutant (*gpa1-5gcr1-5*) grown on B5 media supplemented with low nitrate (12.5 mM KNO_3_) optimal nitrate (25 mM KNO_3_) as per the standard B5 media composition or high nitrate (30 mM KNO_3_) at 22 °C in a growth chamber. The emergence of radicle was observed every three hours for the next three days (72 h) and total % seeds germinated and time taken for 50% seeds to germinate were used to compare WT and mutants. All of them started germinating around 30 h after soaking and seeds of both the single mutants and wild type were broadly similar at all nitrate doses, both in terms of total germination at 72 h (95–100%) and the time taken for 50% seeds to germinate (Fig. 9). However, the double mutant was sensitive to low nitrate level (12.5 mM) on both counts. It had significantly lower level of total germination (80%) and also significantly slower germination rate, as the time taken for 50% seeds to germinate was delayed by 4 h relative to the WT (Fig. 9B).

In order to investigate whether these mutants are affected in the genes encoding nitrate uptake and metabolism, we grew them along with WT in low (12.5 mM) and high (30 mM) nitrate conditions for 14 days, harvested their root tissues and analyzed the expression of known nitrate-regulated genes, nitrate transporter (*NRT1*) and nitrate reductase (*NR2*) by real time PCR. At 12.5 mM nitrate level, the expression of *NRT1* was higher in all three mutants as compared to wild type, whereas *NRT1* expression was reduced at 30.0 mM nitrate (Fig. S7). Considering that *NRT1* is known to be a low affinity nitrate transporter and sensor or transceptor, 12.5 mM nitrate may have been perceived as inadequate due to *GCR1* and/or *GPA1* mutation, triggering higher expression of *NRT1*, which was not the case at 30.0 mM nitrate. This is consistent with our previously reported role for Gα signaling in N-response and nitrate reductase expression/activity^5,17^. Accordingly, the perceived nitrate-limited condition in both the single and double mutants also explains the observed down-regulation of nitrate reductase (*NR2*) transcript level at 12.5 mM nitrate but not at 30 mM nitrate, except in the *gpa1-5* mutant (Fig. S7). We also demonstrate for the first time that *gcr1-5* mutant shows altered dose-dependent differential N-response for both *NRT1* and *NR2* gene expression, implying GCR1-GPA1 coupling in N-signaling.

## Discussion

It is well recognized that heterotrimeric G-proteins play important roles in several plant processes, despite the limited diversity of their components^24^. For example, all the functions of the Gα subunit were previously attributed to GPA1 in *Arabidopsis*, till it was shown that a few of these functions are attributed to XLGs^3,55^. The existence of multiple γ subunits necessitated the classification of downstream signalling partners/pathways in *Arabidopsis*^56^. Normally, this would also be expected for molecules upstream of G-proteins, as their diversity facilitates the perception, discrimination and transduction of diverse signals. Instead, they were viewed from a predominantly all-or-none approach that initially relied only on GPCRs^14^ and subsequently relied only on RGS^20^, arguing explicitly that only one of the two possibilities can exist^24^ till recently. We have provided the first evidence against such exclusive approach using parallel functional genomic analyses of mutants of *Arabidopsis GCR1*^16^/*GPA1*^17^ from a gene discovery perspective. We showed there by Venn selection that 30% of all *GCR1-*responsive genes and 57% of all *GCR1*-regulated processes were similar to those of GPA1, though there were also many that did not overlap with those of *GPA1*. This was by far the most compelling indication, not only in favour of the GCR1-GPA1 partnership, but also in favour of its possible coexistence with other alternative partnerships (GCRx-GPA1, GCR1-GPAx, non-GCR-partnership with GPA1 or GCR1 partnership with a non-G-protein).

In this study, we extended this approach further by microarray analysis of a double-mutant generated from the confirmed single mutants of *GCR1*^16^ and *GPA1*^17^ in *Arabidopsis* to further confirm the genes/processes co-regulated by GCR1-GPA1 partnerships, as well as to predict other possible partnerships based on the observed responses. This double mutant (*gpa1-5gcr1-5*) is different from the only other double mutant reported so far^11^, not only because it is in a different ecotype, but also with respect to the specific loci of mutations in their single mutant parents we generated and used for crossing, as described earlier for *gpa1-5*^17^ and *gcr1-5*^16^. The double mutant was found to be phenotypically similar to the previously published double mutant^11^ as well as closer to the *gpa1-5* parent (Fig. 1B–E)^17^, further confirmed by hierarchical clustering (Fig. 2). We found 656 DEGs in the double mutant spanning all 5 chromosomes, with nearly equal proportion of up/down-regulated genes. Nineteen of these genes (10 up-regulated and 9 down-regulated) have been verified by qRT-PCR (Fig. 3) and a larger list of the top 10 DEGs is given in Table 1. Functional annotation and MapMan pathway enrichment analysis showed that these DEGs were involved in many pathways such as response to external stimulus, primary and secondary cell wall modulation/biosynthetic processes, plant immunity, secondary metabolism, nitrogen signaling and light signaling among others.

The genes/processes identically regulated in all 3 mutants can be best explained by GCR1 and GPA1 working together in the same G-protein signalling pathway, though co-regulation by convergence of independent pathways cannot be ruled out, till the clinching biochemical evidence for the functional coupling of GCR1 and GPA1 is obtained. On the other hand, independent signalling pathways of GCR1 and GPA1 provide the most plausible explanation for the regulation of the 51 additional genes in the double mutant shared only with the *GCR1* mutant, as well as for the 150 additional genes shared only between the double mutant and the *GPA1* mutant. At least some of these DEGs in the double mutant common to either of the two single mutants (but not both) belong to the same process categories including stress, response to stimulus, transcription, etc. that are shared by all three mutants. This means that even when GCR1 and GPA1 follow independent pathways involving other partners to regulate different genes, some of them seem to achieve similar regulatory outcomes at the process level. This is indeed the best explanation for 195 unshared genes from the *GCR1* single mutant and 140 unshared genes from the *GPA1* single mutant belonging to 41 shared processes in the double mutant. These processes include response to stress, cell wall modification, development, hormone response, etc.

To understand the functional association of DEGs and associated processes regulated by GCR1 but independent of GPA1, we compared the list of DEGs and found that 51 DEGs in the double mutant shared only with the *gcr1-5* mutant and not with the *gpa1-5* mutant constitute about 44% of the 115 total DEGs shared between them (as the remaining 64 are common to all 3 mutants). Their identical pattern of regulation in the *gcr1-5* mutant and the double mutant clearly indicates that the effects of *GCR1* mutation are carried over to the double mutant but *GPA1* mutation has no effect on these genes, either in the *gpa1-5* mutant or in the double mutant. The best explanation for this is that GCR1 regulates these genes through some other partner, which may be another GPA-like isoform that is yet to be identified, or the Gβ and/or Gγ, RGS, XLG components of heterotrimeric G-protein complex, or through a totally different, non-G-protein signalling mechanism. While testing these possibilities is beyond the scope of the current study, it does offer a list of genes regulated through such a partnership as a starting point to test these hypotheses.

The significant overlap of DEGs (150 using stringent cut-offs) between *gpa1-5*^17^ and the double mutant suggests their regulation via GPA1 but independent of GCR1 function. Even though they form a minority of the 656 DEGs identified in the double mutant, they constitute 70% of all the 214 DEGs shared between *gpa1-5* and the double mutant (as the remaining 64 GPA1-regulated genes are shared between all 3 mutants). Their huge overlap and identical differential regulation explains the sheer predominance of the effects of *GPA1* mutation in the double mutant, in terms of the 92% similarity in the 79 processes to which their shared DEGs belong as well as their phenotypic traits.

We detected a higher number of DEGs (including exclusive DEGs) in the double mutant than in either of the single mutants. The exclusive biological processes in all three datasets revealed overrepresentation of cell wall modification/biogenesis/organization, response to light intensity, flavonoid biosynthetic and metabolic processes among others in the double mutant; hydrogen peroxide metabolic and catabolic processes in the *gpa1-5* mutant and response to starvation, phosphate starvation and nutrient levels in the *gcr1-5* mutant (Fig. 7A). This clearly suggests that modulation of cell wall composition requires both GCR1 and GPA1 function. MapMan pathway analyses also showed significant enrichment of cell wall associated DEGs in the double mutant as compare to either of the single mutants (Fig. 7B).

PPI network analysis yielded 7 molecular complexes/sub-clustered genes, of which sub-cluster 1 revealed light regulated transcription factors HY5, HFR1, COP1, MYB18 and HFY1. Out of them, HY5 and HFR1 acts downstream of phytochrome A (phyA) mediated signaling and regulate phyA-responsive gene expressions in *Arabidopsis*. HY5 and HFR1 both are positive regulators of phyA signaling and interact with COP1 E3 ligase, which is negative regulator of photomorphogenesis^57^. HFR1 was up-regulated in the double mutant, which suggests that GPA1 and GCR1 may regulate these molecular complexes through HFR1 function and accordingly their associated phenotypic traits and biological responses. We identified another important hub (Fig. 8C) involved in nitrate (N) response regulation in *Arabidopsis*. KIN10 and KIN11 show significant homology with human adenosine monophosphate-activated protein kinase (AMPKα1). It has been shown that loss of KIN10 and KIN11 function reduces mutant sensitivity to N level^58^. Further, the circadian clock-dependent activities of these kinases are regulated by N level and control the flowering time in *Arabidopsis*^58^. Though KIN10 and KIN11 were not identified as DEGs in our mutants but we detected AKINBETA1 (5′-AMP-activated protein kinase beta-2 subunit) as an up-regulated DEG, which is interacting with both KIN10 and KIN11 to constitute molecular complexes (Fig. 8C). This leads to a testable hypothesis that both GCR1 and GPA1 control the N-regulated flowering time via modulating KIN10, KIN11 and associated molecular complexes in plants. The role of HY5 has been established as phloem mobile signal that enhances the nitrate uptake from roots^59^. The NIN-like protein 8 (NLP8), a transcription factor and positive regulator of nitrate signaling, is essential for nitrate-regulated seed germination in Arabidopsis^53^. Our physiological data on the sensitivity of seed germination to low nitrate in the double mutant (Fig. 9) further support the involvement of G-protein signaling^17^ as a regulator of nitrate response. Our molecular evidence on the differential transcript accumulation of the low affinity nitrate transporter/transceptor (*NRT1*) and nitrate reductase (*NR2*) in the root tissues of single and double mutants at low N (Fig. S7) confirms the role of *GCR1* and *GPA1* coupling in nitrate signaling. Further examination of G-protein signaling in N response and NUE is in order, in view of these and earlier studies^17,60^ in this regard. Hormones control developmental and defense responses by orchestrating cellular pathways. GO and MapMan analyses showed many DEGs associated with hormone biosynthesis as well as signaling (Fig. 4 Table S3). The DEGs involved in auxin and ethylene biosynthesis were overrepresented among other hormonal pathways (Fig. 4). We also detected the auxin-related molecular complexes comprised of indole-3-acetic acid inducible 31 (IAA31), auxin response factor 16 (ARF16) and indole-3-acetic acid inducible 5 (AA5). IAA3 was down-regulated in the double mutant, but how GPA1 and GCR1 coordinate these hormonal responses involving IAA3 is yet to be discovered. The GO terms for response to stimulus and biotic stresses belong to highly enriched biological processes (Fig. 2B). MapMan analyses also highlighted the biotic stress as a major pathways/bin (Fig. 4). Further sub-clustering of PPI networks showed that regulatory protein (NPR1), NPR1-like protein 3 (NPR3), and AHBP-1B (bZIP transcription factor family protein) are involved in the formation of molecular complexes (Fig. S6). NPR1 and NPR3 are salicylic acid receptors and AHBP-1B interacts with these receptors to modulate the expression of PR genes in *Arabidopsis*^42^. AHBP-1B was up-regulated in the double mutant, which suggests that combined function of GPA1 and GCR1 modulate plant immunity. Further investigation is needed to understand the mechanism of immune regulation by co-functionality of GPA1 and GCR1 in *Arabidopsis*.

## Conclusions

This is the first comprehensive transcriptome analysis of *gpa1-5 gcr1-5* double mutant that goes beyond abiotic stress^6^, and provides compelling genetic evidence to our earlier findings based on the single mutants^16,17^ on: a) the role of GCR1 in G-protein signalling and b) the combinatorial involvement of GCR1 and/or GPA1 in regulating different gene sets and c) specific evidence of *GCR1*-*GPA1* coupling in mediating nitrate response. Our analysis reveals the genes/processes identically regulated in both single and double mutants, providing the strongest genetic evidence thus far for GCR1-GPA1 coupling, at least in *Arabidopsis*. They include cell wall composition/processes, plant immunity, nitrogen signaling and biosynthesis of isoprenoids, stress, development and nutrient transport, among others. PPI network analyses and MCODE sub-clustering led to the identification of seven hub key genes, which are regulated by coupling of GPA1 and GCR1. Our comparative analysis of the mutants also reveal the genes/processes that are affected only by either GPA1 or GCR1 in the single mutants but not in the double mutant, providing a starting point to find their other signaling partners, including, but not limited to other isoforms of GCR/GPA. Most importantly, we identified genes uniquely regulated in the double mutant but not in any of the single mutants, though the processes to which they belong may not be so exclusive.

## Methods

### Isolation of double mutant

The *gpa1-5 gcr1-5* double mutants were obtained by crossing the *gcr1-5* mutant^16^ to *gpa1-5* mutant^17^. First, a number of homozygous *gpa1-5 gcr1-5* individuals were identified among the F_2_ progeny due to their characteristic phenotype i.e. enlarged roundish rosette leaves under the short-day growth conditions. Second, these individuals were subjected to the PCR analyses to test for the absence of the *GPA1* and *GCR1* gene copies. Predicted *gpa1-5 gcr1-5* double mutant individuals were allowed to self-pollinate, and homozygosity for both gene mutations were verified using S_2_ segregation analyses on drugs (BASTA and Kanamycin).

### Growth conditions and phenotypic characterization

Both the mutant and wild type seeds were surface-sterilized using 70% ethanol and washed thrice with autoclaved ultrapure water and stratified at 4 °C for two days on half-strength B5 agar plates. The plates were incubated in a growth chamber at 22 ± 1 °C with a light intensity of 150 μM sec^−1^ m^−2^ and a photoperiod of 16:8 (light:dark). Ten days old plants were transferred to 3.5 cm pots containing a mixture of soilrite and vermiculite (1:1), supplemented with full-strength B5 media and regularly watered using sub-irrigation. The plants were used for the measurement of phenotypic characters throughout their life cycle.

### RNA isolation and microarray analysis

Total RNA was isolated from 23 days old whole plants as described previously^16^. RNA samples were analyzed for quality, quantity and suitability for microarray using Nanodrop spectrophotometer and Bioanalyzer (Agilent Technologies, Santa Clara, USA). The same RNA preparations were also used for confirming the knockout mutants using RT-qPCR with gene-specific primers. The Cy3 labelled cRNAs from independent biological duplicates of the wild type (Ws2) and *gpa1-5gcr1-5* double mutant were subjected to microarray analysis using Agilent 8 × 60k *Arabidopsis* arrays (AMADID 037661) as described^6^. Overall the microarray images were clean, with uniform intensity and very low background noise. The data were extracted using Feature Extraction 10.7 software (Agilent Technologies) and normalized using the recommended ‘Per Chip and Per Gene Normalization’ feature of GeneSpring GX Version 11.5. The correlation coefficients of replicates were obtained by principal component analysis. Log2fold change value of 1.0 and p-value of 0.05 was used as a cut-off for differential-regulation. The Benjamini Hochberg FDR procedure at a cut-off value of p ≤ 0.05 was used for multiple testing corrections. Area-proportional Venn selections were done using the DEG lists in the *gpa1-5*, *gcr1-5* and the double mutants using the online software (http://bioinforx.com/free/bxarrays/venndiagram.php).

### Functional classification/meta-analysis of DEGs

The DEGs were assigned gene ontology terms according to the TAIR 10 database^61^. The DEG lists were subjected to enriched GO categorization using AgriGO2.0 with default settings. The DEGs were mapped into various pathways (bins) using MapMan tool. The coloured boxes in each bin represent the DEGs log2FC values. Further, pathway analysis of the DEGs was done to obtain the list of changed pathways using plant MetGenMAP, which takes AraCyc as the background. Differentially expressed transcription factors were compared with the Plant Transcription Factor Database (plantTFDB ver 2.0).

### Data validation using qPCR

A few DEGs were selected from microarray data for its validation based on their roles in different biological processes. The genes were selected in a manner such that at least two up-regulated and two down-regulated genes figured in each of the described biological category. The RT-qPCR was carried out using 1.0 μl of 1:50 diluted cDNA, reverse transcribed form 5 μg of DNase treated RNA. PCR amplifications were performed in 20 μl reactions using the KAPA SYBR® FAST Master Mix (2X) Universal (Kapa Biosystems, USA) with 100 nmoles of each gene-specific primer in Stratagene Mx3000P (Agilent technologies). The amplifications were carried out using biological triplicates, two of which were the same as those used for microarray. Serial dilutions were used to check for primer efficiency and only those primers that worked at 100 ± 10% efficiency were used for all qPCR analyses. The specificity of primer pairs was confirmed by melting curve analysis of the amplicons. *Actin2* (*ACT2*) was used as an internal control for normalization. Quantification of the relative changes in gene expression was performed by the standard curve method.

### Construction of PPI networks and sub-clustering analyses

The exclusive DEGs identified in the double mutants were used to retrieve the interactors from STRING (https://string-db.org/) and BioGRID (https://thebiogrid.org/) databases. The experimentally validated interactions were considered to create the PPI networks and DEGs were mapped using Cytoscape version 3.0. Molecular complex detection (MCODE) plugin was used to perform the sub-clustering of the networks and identification of the molecular complexes associated with various pathways.

### N-responsive seed germination assay

Seeds of *Arabidopsis* wild-type (Ws2) and all three mutants (*gpa1-5*, *gcr1-5*, *gpa1-5gcr1-5*) were surface-sterilized using 70% ethanol for 5 minutes and subsequently washed 5 times with ultrapure water. The stratification of seeds was carried out at 4 °C in total darkness for 48 h to facilitate uniform germination. These stratified seeds were placed on 1X B5 agar plates supplemented with different concentrations of KNO_3_ [optimal nitrate as per standard B5 media composition (25 mM), low nitrate (12.5 mM) and high nitrate (30 mM)]. Plates were transferred to the growth chamber maintained at 22 ± 1 °C with photoperiod (12 h of light/dark period). After 12 h, we examined the seed germination at every 3 h till 72 h.

For qPCR analyses, surface sterilized and stratified seeds of the wild type and all three mutants were grown in B5 medium containing 12.5 and 30 mM KNO_3_ at 22 °C ± 1 in a growth chamber. Root tissues (~100 mg) were used to extract their total RNA using Trizol (Invitrogen, USA) as described by the manufacturer. DNase I treated total RNAs were transcribed into cDNAs using RevertAid first strand cDNA synthesis kit (Thermo Fisher Scientific). The qPCR reaction was performed using KAPA SYBR FAST Master Mix (2x) Universal (Kapa Biosystems, USA) or Brilliant III Ultra-Fast SYBR Green QPCR Master Mix on Agilent MxPro3000P machine. The comparative C(T) method was used for relative quantitation of the transcript and the expression of the genes was normalized using actin as a reference gene.

## Supplementary information

Supplementary Tables

Supplementary Figs

## Data Availability

GEO accession number GSE 40217 (GSM 988511 and GSM 988512).
