# Supplementary material for: GCR1 and GPA1 coupling regulates nitrate, cell wall, immunity and light responses in Arabidopsis
Source: Sci Rep. 2019 Apr 9;9:5838. doi: 10.1038/s41598-019-42084-2 (PMC6456573; doi:10.1038/s41598-019-42084-2)
Supplement: Supplementary file 2 — Supplementary Figs [file 41598_2019_42084_MOESM2_ESM.docx]

**GCR1 and GPA1 coupling regulates nitrate, cell wall, immunity and light responses in *Arabidopsis***

**Navjyoti Chakraborty, Kostya Kanyuka, Dinesh Kumar Jaiswal, Abhineet Kumar, Vivek Arora, Aakansha Malik, Neha Gupta, Richard Hooley and Nandula Raghuram**

**
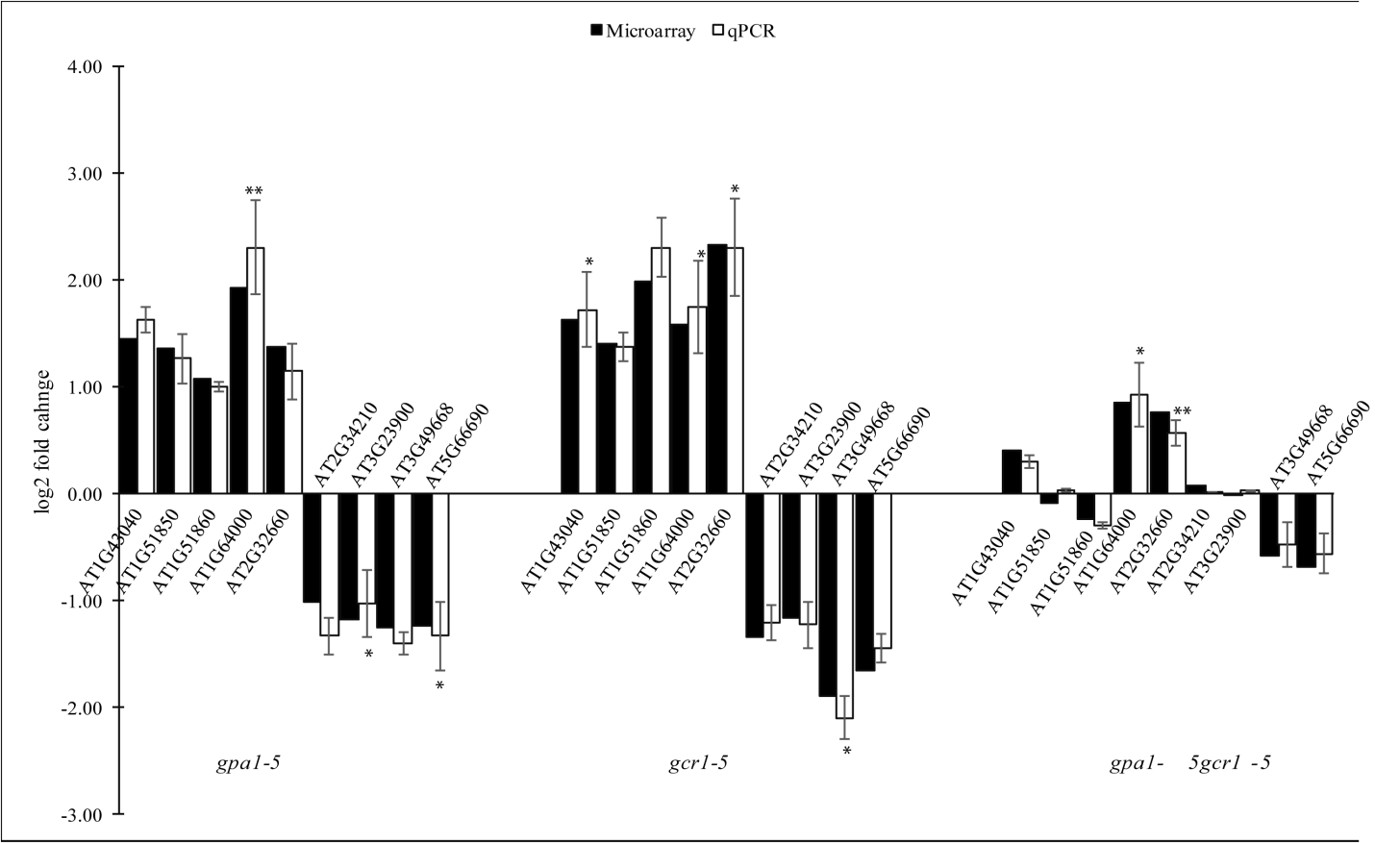
**

**Fig. S1.** qPCR validation of a few DEGs common only to the single mutants, *gpa1-5* and *gcr1-5*. The experiment was carried out using biological duplicates and technical triplicates and the values are presented as log2FC ± SE (*P<0.05, **P<0.01 according to unpaired t-test using GraphPad Prism 6.0).

**
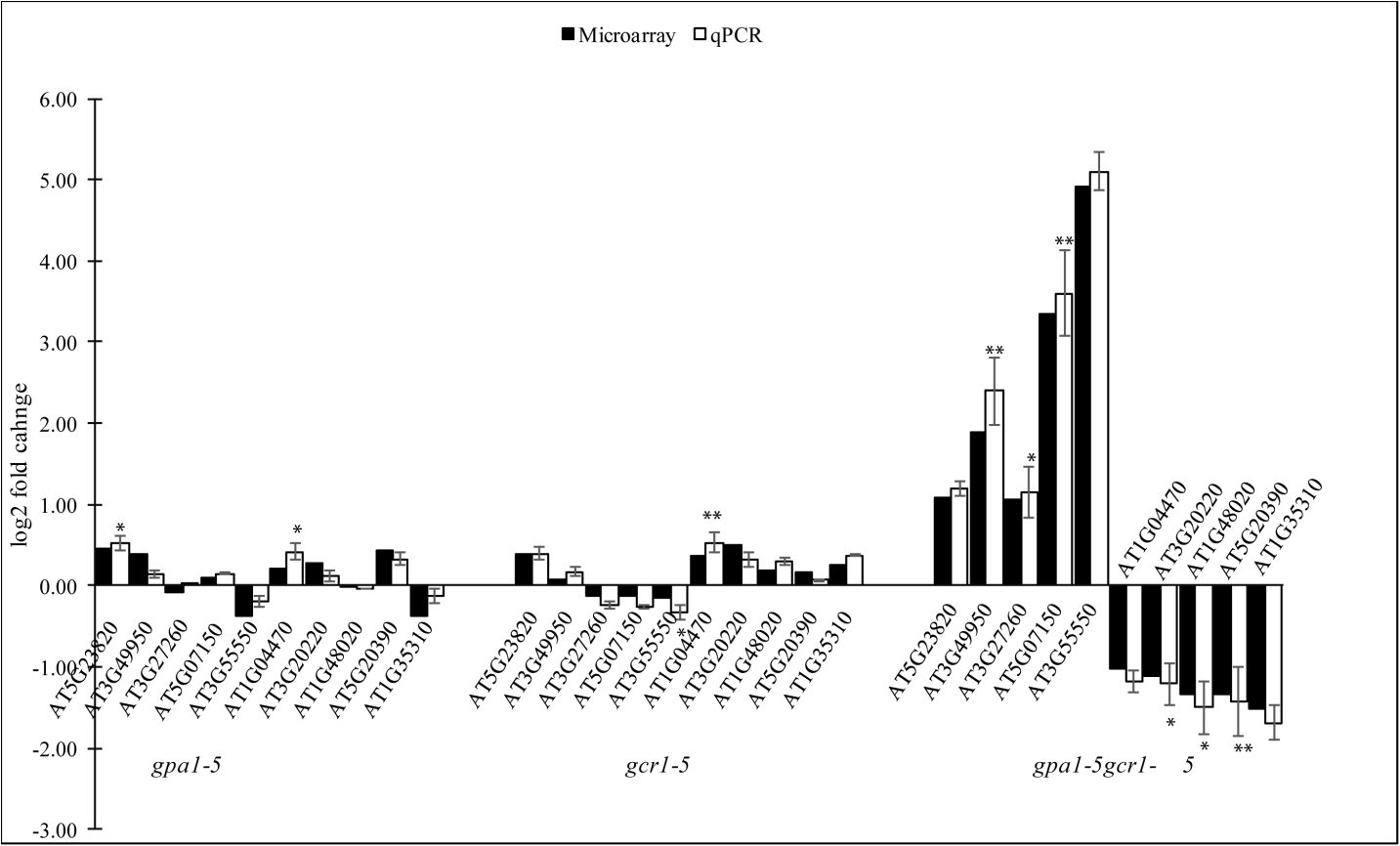
**

**Fig. S2.** qPCR validation of a few DEGs unique to the double mutant, *gpa1-5gcr1-5*. The experiment was carried out using biological duplicates and technical triplicates and the values are presented as log2FC ± SE (*P<0.05, **P<0.01 according to unpaired t-test using GraphPad Prism 6.0).

**
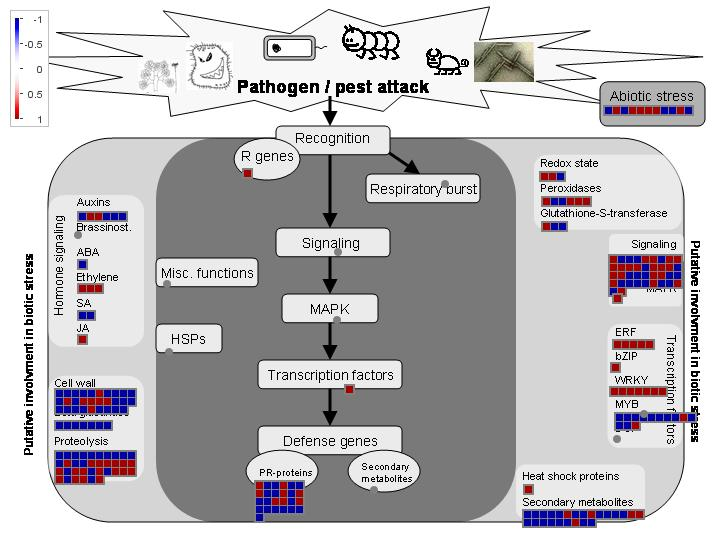
**

**Fig. S3.** DEGs identified in the *gpa1-5gcr1-5* double mutants mapped to biotic stress pathways using MapMan. The double mutant DEGs assigned into different sub-categories of biotic stress bin. The red box represents the up-regulated genes; blue box represents the down-regulated genes. This figure was generated using MapMan software under a CC BY open access license (https://creativecommons.org/licenses/by/4.0/).

**
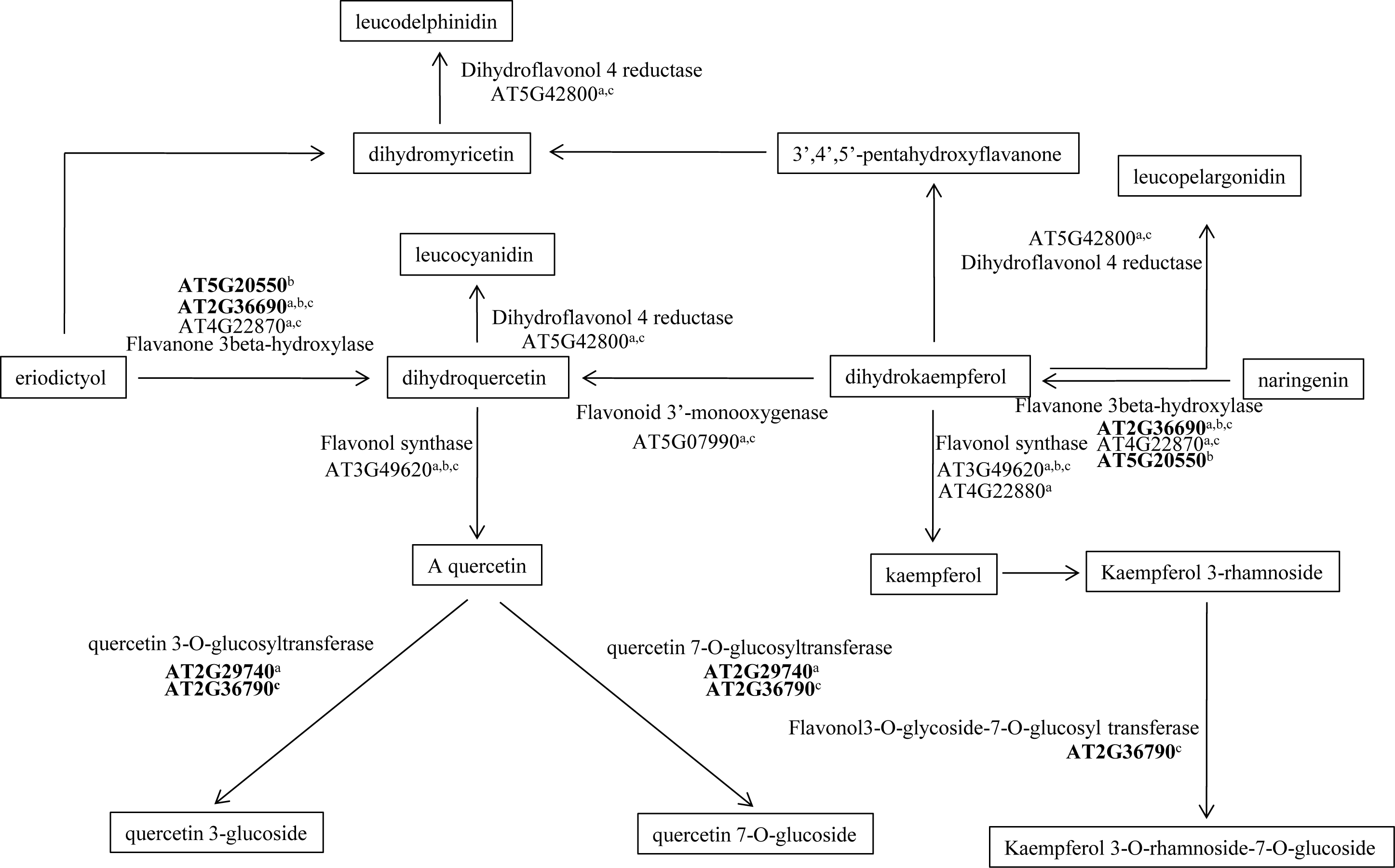
**

**Fig. S4**. Sub-pathway analyses of flavonoids and derivatives (modified figure from Chakraborty, et al., 2015, PLoS One; Chakraborty, et al., 2015, Plant molecular biology). It clearly shows that more genes are differentially regulated in the double mutant, *gpa1-5gcr1-5* than the single mutants, *gpa1-5* and *gcr1-5*. Genes marked in bold are up-regulated while the rest are down-regulated. ^a^ DEG in *gpa1-5*. ^b^ DEG in *gcr1-5*. ^c^DEG in *gpa1-5gcr1-5*.


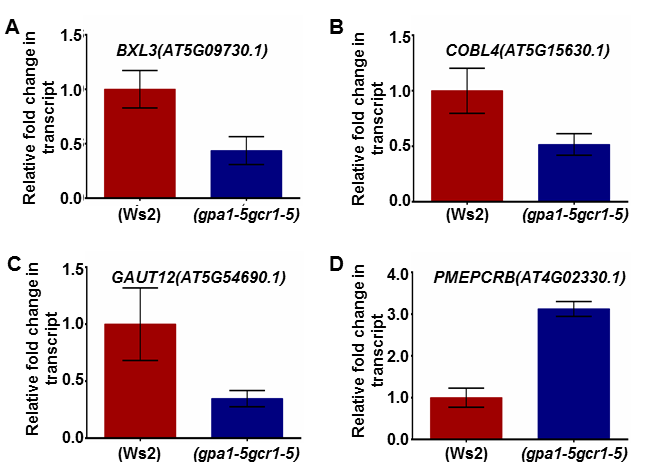


**Fig. S5**. Validation of differentially expressed genes (DEGs) associated with cell wall in *gpa1-5 gcr1-5* double mutant. The relative transcript abundance of four DEGs related to cell wall was analyzed by qPCR in the *gpa1-5 gcr1-5* double mutant using Ws2 as wild type. The reaction was performed in Aria Mx real-time PCR system (Agilent technologies) and actin-2 was used as internal control gene to normalize the expression of the candidate genes. The comparative C(T) method was used to determine the relative expression of genes and error represents ± SE. *BXL3*, Beta-Xylosidase 3; *COBL4*, COBRA-Like 4; *GAUT12*; Galacturonosyl Transferase 12; *ATPMEPCRB*, pectin methylesterase.

**
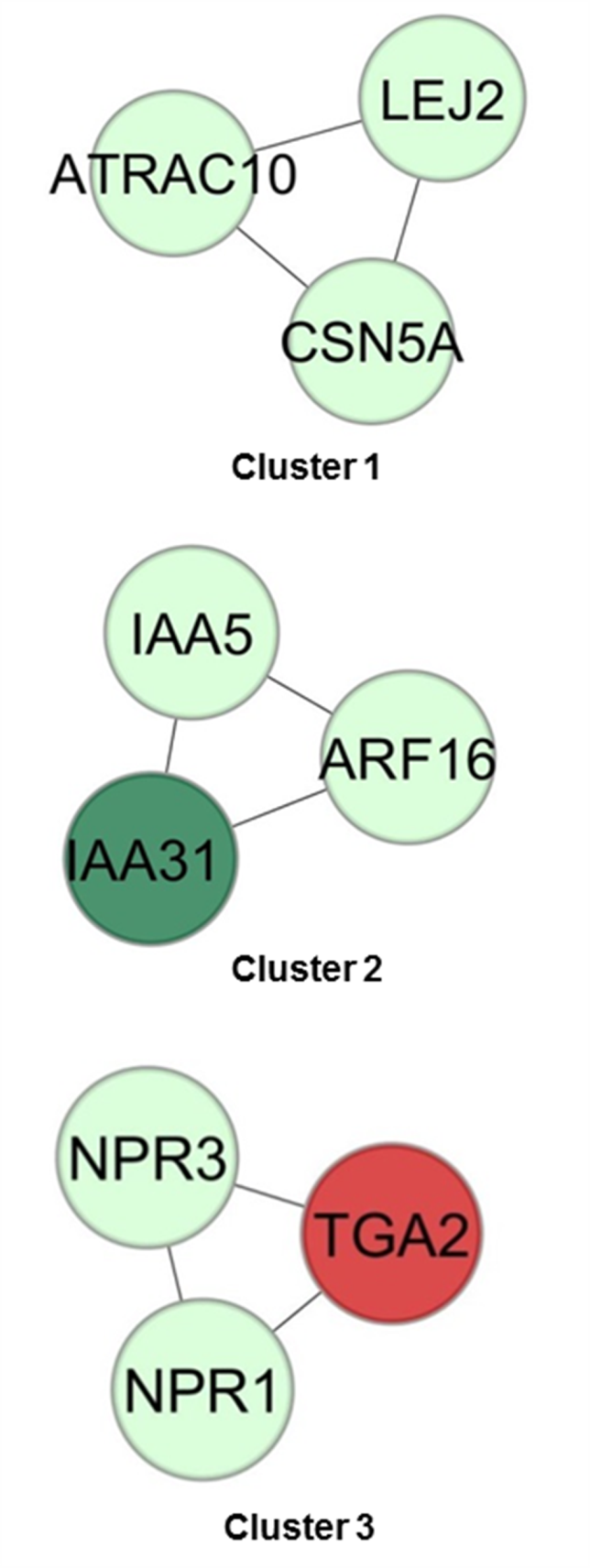
**

**Fig. S6**. Sub-clusters/molecular complexes associated with exclusive DEGs identified in the *gpa1-5gcr1-5* double mutant.


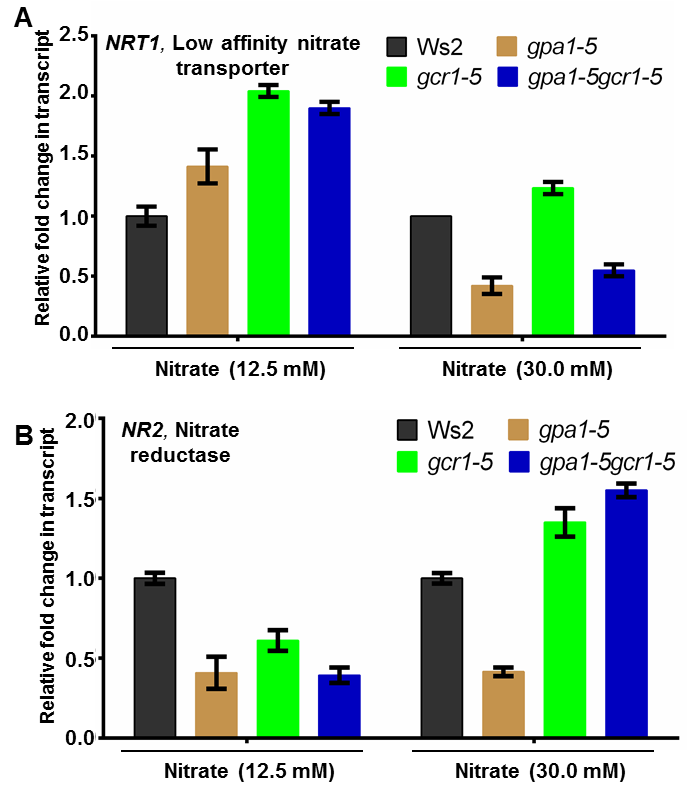


**Fig. S7**. Transcript abundance of nitrate transporter and nitrate reductase genes in the roots of wild type, single and double mutants of GPA1 and GCR1grown in 12.5 and 30 mM nitrate. Seeds of wild type (Ws2), single mutants (*gpa1-5* and *gcr1-5*) and double mutant (*gpa1-5* *gcr1-5*) were grown for 14 days in B5 medium containing 12.5 and 30 mM nitrate at 22°C in a growth chamber. Total RNA was isolated from the roots of mutants and wild type seedlings and qPCR was performed for low affinity nitrate transporter (*NRT1*) and nitrate reductase (*NR2*). The expression of *NRT1* and *NR2* was normalized using actin as a reference gene and relative quantification of transcripts from 3 replicates are shown with ± SE.
